# Treatment Outcomes of Vulvar Cancer: Our Experience From a Tertiary Care Center in Eastern India

**DOI:** 10.7759/cureus.74236

**Published:** 2024-11-22

**Authors:** Tashbihul Azhar, Shiv Rajan, Naseem Akhtar, Vijay Kumar, Sameer Gupta, Jagjit Pandey, Rebba Ephraim, Pravin Kumar

**Affiliations:** 1 Surgical Oncology, All India Institute of Medical Sciences, Patna, Patna, IND; 2 Surgical Oncology, King George’s Medical University, Lucknow, IND; 3 Surgical Oncology, King George's Medical University, Lucknow, IND

**Keywords:** chemotherapy, female vulvar cancer, flap necrosis, human papillomavirus, infection, radiotherapy, seroma, squamous cell carcinoma, vulva

## Abstract

Introduction: The vulva is the external genitalia in females. Vulvar carcinoma is a rare entity, with squamous cell carcinoma as the most common histology while basal cell carcinoma, extra-mammary Paget’s disease, and melanoma are still rarer subtypes. Treatment often comprises a multidisciplinary approach with surgery being the mainstay, followed by adjuvant therapy, depending upon pathological stage and other factors. Neoadjuvant treatment is considered in locally advanced cases where upfront surgery would not achieve adequate margin or organ preservation (urethra and anal canal). Due to the rarity of the disease and initial presentation at advanced stages in third-world countries, it is important to have strict treatment protocols with identifications of various prognostic factors and regular timely follow-up, which would, in turn, improve treatment outcomes. Here, we are presenting our institutional retrospective cohort spanning over eight years.

Methods: This is a retrospective descriptive study done in the department of surgical oncology of a tertiary care cancer center in India from January 2016 to April 2024. A total of 21 patients with histologically proven diagnoses of vulvar cancer were included in the analysis. Stage of disease, treatment modalities used, and disease outcomes in terms of survival were tabulated. Statistical analysis was done using SPSS version 17 for Windows (SPSS Inc., Chicago, IL).

Results: The estimated five-year overall survival was ≈71% using Kaplan-Meier analysis. On univariate analysis (testing other factors), using a log-rank test, neither stage nor nodal positivity were significant prognostic factors for overall survival. Given the small number of cases, multivariate analysis was not possible.

Conclusion: In carcinoma of the vulva, treatment should be individualized with multidisciplinary cooperation. In many series, stage, nodal positivity, and extracapsular extension are found to be significant prognostic factors, though our results did not correspond with these data probably due to a small cohort. The paucity of data, especially from India and other developing countries, necessitates the need for more studies, preferably multicentric, keeping in mind the low prevalence of disease.

## Introduction

Among all gynecologic malignancies, vulvar cancer accounts for 2-6%, corresponding to 0.7% of all cancers in women [1]. Squamous cell carcinoma (SCC), the most prevalent subtype, has long been regarded as a disease of postmenopausal women. Over the last 30 years, India has seen a decline in vulvar cancer cases. There is a decrease in incidence from 2.25% between 1984 and 1988 to 0.33% between 2004 and 2008 [2], with median age being 65-70 years. Globally, from 2008 to 2017, the yearly rate of vulvar cancer incidence has been rising by an average of 0.6% with a decline in the median age of presentation during this time [3].

Age, smoking, immunosuppressive diseases, and chronic vulvar skin diseases (e.g., lichen sclerosus, vulvar intraepithelial neoplasia, and erythroplasia of Queyrat) are the main risk factors for vulvar cancer; however, the rising incidence of human papillomavirus (HPV) infections is likely to be blamed for the affliction of even younger, sexually active women by this disease [4-7].

The aim of this study was to evaluate the oncological outcomes in patients with vulvar cancer treated with curative intent and to compare our results with other published series.

## Materials and methods

Materials

This is a retrospective descriptive study conducted in the department of surgical oncology of a tertiary cancer care center (Eastern India) from January 2016 to April 2024. Included in this analysis were histologically proven 21 patients of vulvar cancer. We tallied the disease stage, treatment approaches and modalities, and survival outcomes. Case records were revisited to procure the clinical data. All patients were treated with curative intent and were followed up periodically as per institutional protocol. Preoperatively, all patients had a thorough physical examination, standard radiographs of the chest, necessary blood investigations, and contrast-enhanced computed tomography (CECT) scans of the pelvis and abdomen, including the groin area. We sparingly used magnetic resonance imaging (MRI) in cases of disease's proximity to the urethra and anus. The main lesion was histopathologically documented before surgery. The presence of metastases to the inguinal nodes was confirmed by fine needle aspiration cytology (FNAC) of palpable nodes in the inguinal region. Enlarged pelvic nodes did not warrant FNAC. The evaluation did not include testing for HPV. All management decisions were taken in a multi-disciplinary tumor board.

Surgical options for management included wide excision with adequate margin (WLE), hemi-vulvectomy (HV), and radical vulvectomy (RV) with unilateral or bilateral inguinal node dissections and iliac node dissections if needed.

The RV procedure at our center followed a strict protocol, taking at least 1 cm of margin in all directions and incising all the way to the urogenital diaphragm fascia. Along with the tumor, the labia majora and minora, as well as the clitoris, were removed. While in HV and WLE, the primary tumor was carefully removed taking at least a 1 cm margin all around, while the remaining unaffected portion of the vulva was preserved. No attempt was taken to reconstruct in any case.

Through distinct transverse incisions below the inguinal ligament, inguinal block dissection was executed either unilaterally or bilaterally. If the inguinal nodes tested positive on FNAC or if imaging revealed enlarged pelvic nodes, iliac node dissection was performed.

When indicated, adjuvant external beam radiation was administered in cases where the margin was positive, the margin was too narrow to allow re-excision, more than one node was involved, or there was extracapsular nodal extension regardless of the number of nodes.

In the first year of treatment, patients were followed up with three monthly visits, then six monthly visits in the second year, and subsequently once-a-year visits. Each follow-up was mandated through clinical examination, and further tests as needed. Chest X-ray and CECT of the abdomen and pelvis were done yearly.

Methods

An analysis was conducted on the failure pattern and survival. SPSS version 17 for Windows (SPSS Inc., Chicago, IL) was used for survival analysis using the Kaplan-Meier method. Overall survival (OS) was calculated from the date of diagnosis till death due to any cause. Using the log-rank test in the univariate analysis, the important prognostic variables were examined. Statistical significance was defined as a value below 0.05. Other public data were used to compare the results [6-10].

## Results

During this eight-year span, 21 individuals diagnosed with invasive vulvar cancer were treated with surgery. The patients' ages ranged from 25 to 75 years, with a median of 55 and a mean of 49.31 (Table 1). All patients primarily presented with discharging ulcers associated with itching that had developed over their external genitalia. The most common site of lesion was labia majora (77.27%), followed by labia minora. Clitoris was also involved in 4.5% of patients.

**Table 1:** Mean and median age of patients in different series.

Study	Study period	Total number of patients	Mean age (years)	Median age (years)	Age range (years)
Le et al. [11]	1980-2004	58	-	71.3	28.3-90.9
Bafna et al. [12]	1996-2000	37	54.7	60	24-80
Sharma et al. [13]	1998-2005	60	-	63	24-92
Hampl et al. [14]	1998-2007	102	57	-	18-93
Eke et al. [15]	1998-2009	11	61.2	-	54-79
Soliman et al. [16]	2002-2009	34	-	56.5	23-86
Present series	2016-2024	21	49.31	55	25-71

Types of surgical interventions

The various surgical procedures including lymph node dissections are listed in Table 2. No lymphadenectomy was performed in five out of 21 individuals. Among these five patients, one had undergone primary closure after wide local excision for verrucous lesions and experienced nodal recurrence three years later, which necessitated bilateral ilioinguinal block dissection, followed by adjuvant radiation therapy (RT). Out of the remaining four patients, one had a pure verrucous lesion, two had microinvasive carcinoma with an invasion depth of less than 1 mm, and one was an older female (>75 years old) with a small primary tumor and other health issues, hence no nodal dissection was performed on her.

**Table 2:** Surgical procedures.

Surgery for primary tumor	Number of patients	Node dissection		
		Unilateral	Bilateral	None
Radical vulvectomy	1	0	1	0
Hemi vulvectomy	1	0	1	0
Wide local excision	19	6	8	5
Total	21	6	10	5

In one patient (out of the entire cohort) posterior pelvic exenteration was done with permanent end colostomy.

The final histopathological examination revealed two cases of verrucous carcinoma and two cases of microinvasive disease. The rest cases were reported as invasive SCC. R0 resection was achieved in all cases. Following nodal dissection, two out of 16 patients (12.5%) reported positive for nodal metastases, both unilateral with no extracapsular extension (ECE). The remaining patients reported negative for nodal disease in the final histopathology.

Complications

No perioperative mortality occurred within 30 days. Postoperative complications primarily resulted from nodal dissection, Clavien-Dindo I-III (Table 3). The most common and troublesome complication was wound gaping following inguinal nodal dissection caused by partial flap necrosis, which occurred in 50% of cases. Debridement of the necrosed area and subsequent secondary suturing led to its salvage.

**Table 3:** Postoperative complications.

Complication	Frequency	Percentage
Seromas requiring serial aspiration	4/16	25%
Flap necrosis	8/16	50%
Wound infection	6/16	37.5%
Deep vein thrombosis	0/16	0%

Of the three patients who were eligible for adjuvant radiation, two successfully completed the radiotherapy; however, one patient did not comply and defaulted. Surprisingly, the patient who withdrew from adjuvant radiation is still alive and has managed to stay disease-free for 43 months. After a median follow-up of 30 months, (range: 3-72 months), eight patients experienced recurrence with one systemic and seven locoregional failure. Out of all locoregional failures, three had nodal failure while four patients had local recurrence. Of the three nodal recurrences, two patients presented with mobile nodes and hence were subsequently treated with ilioinguinal block dissection followed by adjuvant RT. Rest one had a fixed nodal mass, which did not respond well to chemo and radiotherapy, and passed away four months later. Out of four local failures, one patient presented with local and inguinal recurrence at the previously operated site, six months after surgery and adjuvant radiotherapy. However, she succumbed four months later. The second one had a relapse at four months and was salvaged with chemoradiotherapy. She has been disease-free since then and is continuing her regular follow-up. Of the remaining two, one had a recurrence 28 months following the initial surgery and was put on palliative chemotherapy, but unfortunately, she passed away four months later. Periurethral recurrence occurred in the second one, 50 months following initial surgery. She defaulted for any further treatment.

Only one patient developed systemic (lung) metastases and was put on systemic palliative treatment with cisplatin and 5-fluorouracil (5FU).

Survival analysis

**Figure 1:**
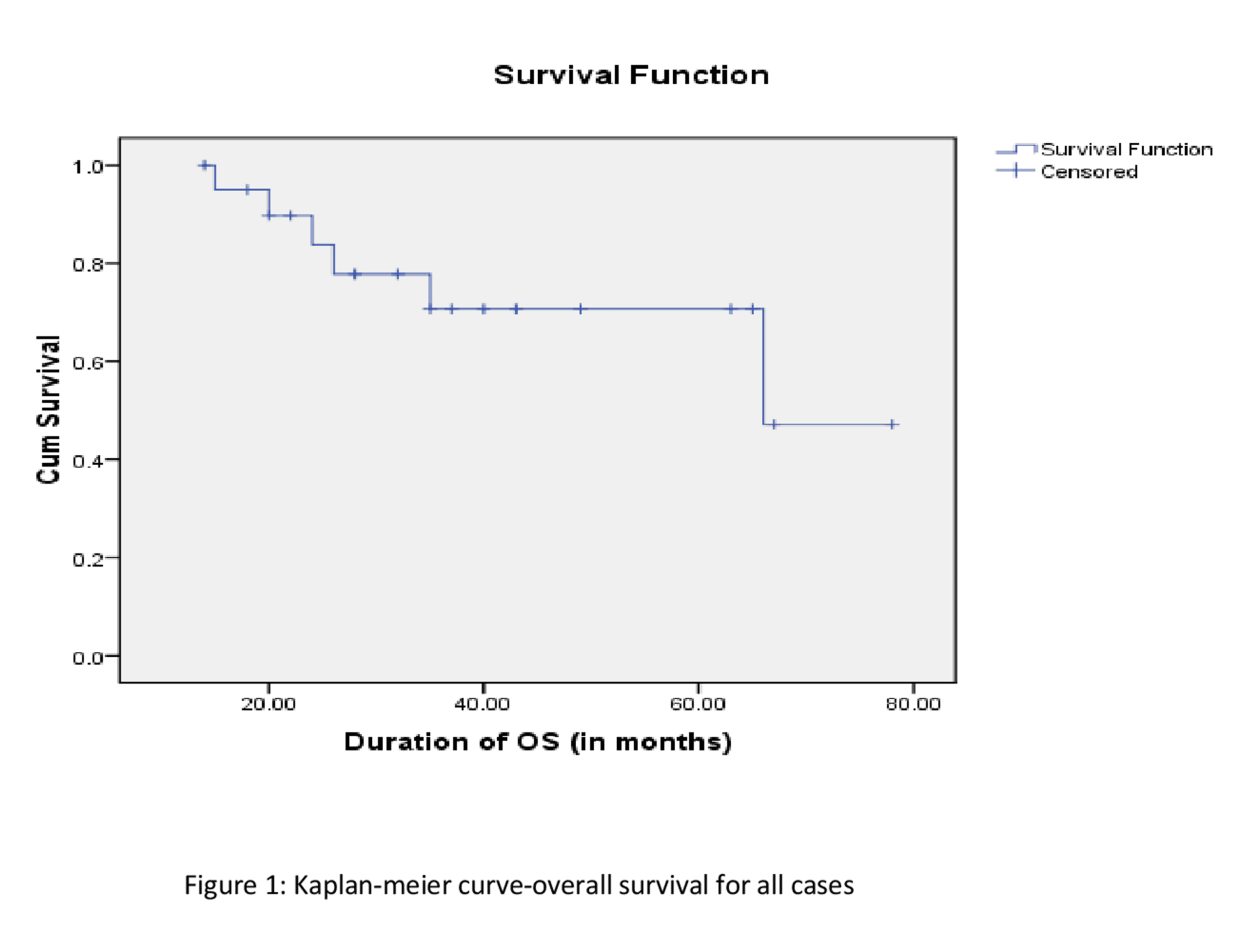
Five-year survival curve. OS: overall survival.

The estimated five-year overall survival (OS) was ≈71% using the Kaplan-Meier analysis (Figure 1). On univariate analysis (testing other factors), using a log-rank test, neither stage nor nodal positivity were significant prognostic factors for OS. Given the small number of cases, multivariate analysis was not possible.

## Discussion

Carcinoma of the vulva is an uncommon malignancy, which usually strikes older women. In our series, 77.27% of lesions were located in the labia majora, 18.18% in the labia minora, and 4.5% involved the clitoris.

Even in this era of organ preservation, radical surgery still has a role in some cases, particularly in third-world countries where presentation is usually late. Nevertheless vast majority of our procedures involved wide excision of lesions with adequate margins with ipsilateral or bilateral inguinal lymph node dissection. After the landmark study from Hacker et al. [6], there is a paradigm shift away from the usual radical en bloc excision of the vulva and inguinal nodes with a single incision to separate incision in the groin and vulvar areas, which resulted in comparable cure rates with less morbidity [6]. Radiation therapy administered after surgery has largely supplanted standard pelvic lymphadenectomy since the publication of Homesley et al.'s study [7].

Margin has always been a contentious issue in many cancers. How much margin is adequate and oncologically safe in cancer vulva is yet not clear, albeit no higher failure rates were observed in 91 patients whose closest tumor margins were 8 mm or more in the fixed specimen, as reported by Heaps et al. [8], who studied the relation between surgical margins and local recurrence. Patients whose margins were less than 8 mm or 8 mm or more did not differ in terms of local recurrence rate, according to research by Groenen et al. [9]. We always try to give at least a 1 cm margin (mean margin was 9 mm) wherever feasible.

An independent predictor of progression-free and overall survival was found to be the total number of nodes harvested following surgery, as observed in a series by Le et al. [11]. They propose to define optimal inguinal nodal dissection using a cut-off value of at least 10 nodes in total for bilateral inguinal block dissection [11]. In our series, the median nodal yield for unilateral and bilateral inguinal block dissection was seven and 16, which indicates optimal dissection, though we did not find the impact of nodes on survival and disease-free interval. The major morbidity of vulvar cancer surgery follows inguinal lymphadenectomy. To determine the presence or absence of regional nodal metastases, some researchers have recently investigated intraoperative lymphatic mapping with frozen section assessment [11,12]. According to an expert panel at the 2008 International Sentinel Node Society Meeting, ''when performed by a skilled multi-disciplinary team in well-selected patients, it is a reasonable alternative to complete inguinal lymphadenectomy'' [13]. As of now, we are yet to practice sentinel node biopsy in vulvar cancer.

Skin flap necrosis occurred in half of the patients in our study (8/16) wherein inguinal nodes were addressed, whereas up to 88.4% of patients in other Indian series experienced significant groin wound dehiscence after lymphadenectomy. When inguinal lymphadenectomy was performed with saphenous vein sparing instead of ligation, a study by Hampl et al. [14] found that the former group had a significantly lower risk of developing lower extremity lymphoedema and phlebitis in the near term; however, no decrease in the risk of seroma or acute cellulitis was noted [15]. However, according to Soliman et al., ''wound complications after inguinal lymphadenectomy are very high, with no single pre, intra, or postoperative factor that could be incriminated, and saphenous vein sparing provided no significant difference in decreasing local complications'' [16].

We do not practice saphenous vein ligations at our facility unless satisfactory lymph node clearance is threatened. The results of a randomized controlled trial were negative for sartorius muscle transposition in a study by Judson et al. [17]; however, we do this all the time because it stops major vessels from being exposed when inguinal wound gaping occurs as a result of skin flap necrosis.

Three patients (13.6%) defaulted for follow-up in our series. Ironically, it is fairly common for Indian ladies to have poor follow-up owing to a number of variables, such as considerable travel distance, poor socioeconomic level, and advanced age [17]. Compared to other series, ours had a slightly greater recurrence rate (Table 4). Despite the fact that stage and lymph node positivity did not have a substantial impact on OS in our series, other Indian series have shown that these two parameters are strong prognostic indicators for OS [14]. The median number of nodes recovered in our series was eight, which exceeds the recommended minimum of six nodes [18]. Nodal status has been identified as the most important prognostic variable in many series and certain variables related to positive nodes (such as ECE) could be critical and further add to poor outcomes [19-22]. Contrary to these findings, surprisingly, there are few studies wherein nodal positivity did not retain its prognostic significance [23,24]. Nevertheless, most of the studies have concluded that the presence of positive lymph nodes has a significant bearing on prognosis [25-29]. In our study, all regional and systemic recurrences occurred within one year while all local recurrences occurred after one year of primary surgery.

**Table 4:** Recurrence in various series.

Series	Study period	Total number of patients	Median follow-up in months	Recurrence in percentage
Groenen et al. [9]	2000-2005	93	31	23
Landrum et al. [10]	1990-2005	175	54.5	13
Le et al. [11]	1980-2004	58	37	29.3
Bafna et al. [12]	1996-2000	37	-	32.4
Sharma et al. [13]	1998-2005	60	23	43
Cheng et al. [26]	1980-2002	100	-	34
Woelber et al. [27]	1996-2003	103	36	13.6
Present series	2016-2024	21	30	38

Limitations of this study

The major limitation of this study is the rarity of the disease, which led to a small cohort. Even at high-volume oncological centers (ours is a high-volume center), the frequency of such patients is low. Long-distance journeys and poor financial conditions of patients further add to non-presentation. Designing proper treatment guidelines demands a larger cohort of patients that can only be achieved with multicentric collaboration.

## Conclusions

Multidisciplinary teams should work together to tailor treatments for carcinoma of the vulva for each patient's unique needs. Researching the effect of different prognostic factors on survival requires a larger cohort that can be followed for an extended period of time. Despite the fact that comprehensive stage, nodal positivity, and ECE are significant prognostic markers in various series, our results did not correspond with those data, perhaps because of the small cohort. Given the rarity of the disease and paucity of data, especially from India and other developing countries, it necessitates the need for more studies, preferably multicentric, keeping in mind the low prevalence. Uniform consensus and guidelines should be derived from those studies regarding organ conservation strategies and morbidity-reducing approaches.
